# Perforation from Ingested Wooden Toothpick: A Colon Diverticulitis Mimicker: Key learning point: Foreign bodies should be considered in the differential when imaging displays bowel inflammation

**DOI:** 10.5334/jbsr.1885

**Published:** 2019-09-12

**Authors:** François Dermesropian, Olivier Dewit, Vassiliki Pasoglou

**Affiliations:** 1 Cliniques Universitaires Saint-Luc, BE

**Keywords:** colic perforation, toothpick, diverticulitis mimicker

## Case

**Figure 1:**
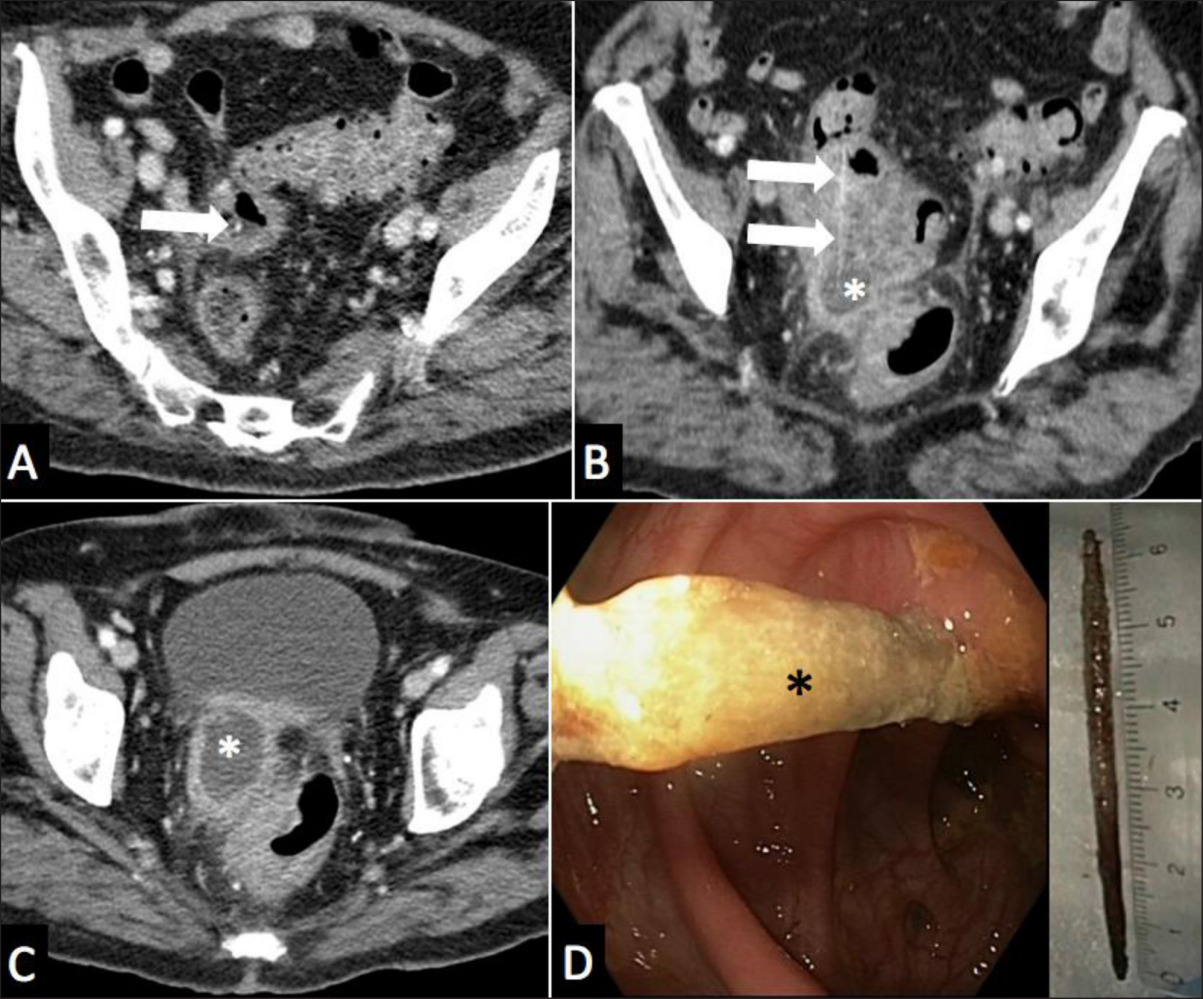


A 79-year-old woman presented to the emergency room with a month history of intermittent hypogastric abdominal pain and fever. Past medical history included diabetes and diverticular colic disease. CT scan with intravenous contrast medium (Figure 1A, B, C) demonstrated a colic diverticulosis with sigmoid wall thickening, extra-digestive abscess of 4.5 cm in diameter (white asterisk), adjacent fat stranding and a 60 mm long, 2 mm thick high density linear structure (arrow) coursing through the colic wall and the abscess. Covered colic perforation and an extra-digestive abscess related to a wooden toothpick were suspected. After antibiotherapy, recto-sigmoidoscopy (Figure 1D) was able to confirm and retrieve the wooden toothpick (black asterisk) embedded through the colic wall. Clinical follow-up was favorable.

## Comment

Perforation can occur in any part of the gastro-intestinal tract as the toothpick can migrate in various anatomic structures. Adequate therapy depends on the localization of the toothpick and the complications. Endoscopic removal is used as the first-line approach. Surgery is reserved for failed endoscopic retrieval and complicated cases such as fecal peritonitis, fistulas, migration to extra-digestive structures and bleeding [1]. This case highlights the fact that tiny or lowly attenuating foreign bodies should be considered in presence of bowel inflammation on imaging, as this may have paramount implication for management.

## References

[1] Sarici, IS, Topuz, O, Sevim, Y, et al. Endoscopic Management of Colonic Perforation due to Ingestion of a Wooden Toothpick. Am J Case Rep. 2017 1; 20(18): 72–75. DOI: 10.12659/AJCR.902004 PMC5270761 28104902

